# Driving factors for the utilisation of healthcare services by people with osteoarthritis in Portugal: results from a nationwide population-based study

**DOI:** 10.1186/s12913-021-07045-4

**Published:** 2021-09-28

**Authors:** Daniela Costa, Ana M. Rodrigues, Eduardo B. Cruz, Helena Canhão, Jaime Branco, Carla Nunes

**Affiliations:** 1 NOVA National School of Public Health, Public Health Research Centre, Universidade NOVA de Lisboa, Avenida Padre Cruz, 1600-560 Lisbon, Portugal; 2 Comprehensive Health Research Centre (CHRC), Universidade Nova de Lisboa, Lisbon, Portugal; 3 EpiDoC Unit, CEDOC, NOVA Medical School, Lisbon, Universidade NOVA de Lisboa, Lisbon, Portugal; 4 Rheumatology Unit, Hospital dos Lusíadas, Lisbon, Portugal; 5 Physiotherapy Department, School of Health, Polytechnic Institute of Setúbal, Setúbal, Portugal; 6 Centro Hospitalar Lisboa Ocidental (CHLO- E.P.E.), Serviço de Reumatologia do Hospital Egas Moniz, Lisbon, Portugal

**Keywords:** Delivery of health care, Osteoarthritis, Socioeconomic factors, Health services, Andersen’s behavioural model of healthcare utilisation

## Abstract

**Background:**

Worldwide, the current management of knee osteoarthritis appears heterogeneous, high-cost and often not based on current best evidence. The absence of epidemiological data regarding the utilisation of healthcare services may conceal the need for improvements in the management of osteoarthritis. The aim of this study is to explore the profiles of healthcare services utilisation by people with knee osteoarthritis, and to analyse their determinants, according to Andersen’s behavioural model.

**Methods:**

We analysed a sample of 978 participants diagnosed with knee osteoarthritis from the population-based study EpiReumaPt, in Portugal. Data was collected with a structured interview, and the diagnosis of knee osteoarthritis was validated by a rheumatologist team. With the Two-step Cluster procedure, we primarily identified different profiles of healthcare utilisation according to the services most used by patients with knee osteoarthritis. Secondly, we analysed the determinants of each profile, using multinomial logistic regression, according to the predisposing characteristics, enabling factors and need variables.

**Results:**

In our sample, a high proportion of participants are overweight or obese (82,6%, *n* = 748) and physically inactive (20,6%, *n* = 201) and a small proportion had physiotherapy management (14,4%, *n* = 141). We identified three profiles of healthcare utilisation: “HighUsers”; “GPUsers”; “LowUsers”. “HighUsers” represents more than 35% of the sample, and are also the participants with higher utilisation of medical appointments. “GPUsers” represent the participants with higher utilisation of general practitioner appointments. Within these profiles, age and geographic location – indicated as predisposing characteristics; employment status and healthcare insurance - as enabling factors; number of comorbidities, physical function, health-related quality of life, anxiety and physical exercise - as need variables, showed associations (*p* < 0,05) with the higher utilisation of healthcare services profiles.

**Conclusions:**

Healthcare utilisation by people with knee osteoarthritis is not driven only by clinical needs. The predisposing characteristics and enabling factors associated with healthcare utilisation reveal inequities in the access to healthcare and variability in the management of people with knee osteoarthritis. Research and implementation of whole-system strategies to improve equity in the access and quality of care are paramount in order to diminish the impact of osteoarthritis at individual-, societal- and economic-level.

**Supplementary Information:**

The online version contains supplementary material available at 10.1186/s12913-021-07045-4.

## Background

Osteoarthritis (OA) is a leading cause of disability worldwide, responsible for 9.6 million years-lived with disability, of which 85% are attributable to the knee joint [1]. The direct costs of knee OA represent 1–2.5% of the GDP of high-income countries, mainly accounted by Total Knee Replacement surgery (TKR) costs. Moreover, the indirect costs can surpass the direct costs, mainly due to work loss or early retirement [2]. In Portugal, 12,4% of the adult population have knee OA [3] and, in 2013, the indirect costs represented 0.4% of the GDP [4]. Portugal has an ageing population, where 80% of the older adults are overweight and 75% of the adult population is physically inactive [5]. This data suggests a progressive and future increase in the prevalence and burden of knee OA, in the same way as in other countries [6].

People with knee OA suffer from chronic pain, fatigue, sleep problems, disability, impaired quality of life and mental health, this limits their participation in social, community and occupational activities [3, 7]. The management of this condition requires integrated multi-disciplinary interventions during the progression of the disease to reduce pain, modify the risk factors and improve function, as there is no known cure for OA [7].

Exercise, maintenance of a healthy body weight, education and self-management strategies are recommended as first-line and core interventions during the disease progression. Pharmacological modalities can help with the symptoms control. TKR should only be considered if the core interventions have failed and, if HRQoL is significantly impaired in selected patients [8, 9], due mainly to the rates of surgical complications and adverse events, associated mortality and low levels of satisfaction with the outcomes [10].

However, data from several countries suggests that the current care for knee OA is heterogeneous and discordant with the quality standards [11]. Medication for pain relief is often the first line treatment prescribed by general practitioners (GP’s) [12], less than 50% of patients are referred to physiotherapy or weight management programs and referrals to the orthopaedic surgeon is often inadequate [11]. Portugal is the country with highest TKR growth rate among OECD countries, where the incidence rate increased by 20% between 2005 and 2011 for patients both above and below 65 years old [13].

International data has shown that overall healthcare utilisation and related costs are significantly higher in patients with knee OA than in the matched non-OA population, even when adjusted for the number of comorbidities [14]. Moreover, the variability in healthcare utilisation can be driven by determinants other than clinical factors, like sex, education level, income, insurance coverage, perceived needs, area of residence and socio-economic status [15].

: According to the Andersen’s Behavioural Model [16], the utilisation of healthcare services levels are influenced by contextual (health organisations provider-related factors and community characteristics, measured at an aggregate rather than individual level) and individual determinants. Contextual and individual determinants may influence health behaviours and outcomes [17]. Individual determinants are classified into the following three domains: 1) predisposing characteristics - demographic variables that influence people to use healthcare services, e.g., age, geographic location, marital status; 2) enabling factors - socio-economic related factors that promote the utilisation of health services, e.g. education level, health insurance; 3) need variables - include risk factors for diseases, individual health states, and experiences of diseases that lead to the utilisation of healthcare services, e.g. self-reported quality of life, functional status or physical activity [15, 16, 18]. In an equitable system, the interventions received would be driven by the clinical needs of the patient [18].

The Portuguese National Health Service (NHS) is a universal coverage, tax-financed system where GP’s are required to act as the gatekeeper to other health services. In addition, there are private health insurance for the general population and, health insurance schemes that cover particular professions, which facilitate access to the private healthcare sector [19].

Currently, there is no published data about healthcare utilisation by people with knee OA in Portugal, and literature with national datasets is scarce. Due to the complexity of this condition, the identification of different profiles of healthcare services utilisation and its determinants is critical to identify needs for improvement at individual and system level and, to develop interventional strategies to mitigate these needs. The aim of this study is to explore different profiles of healthcare services utilisation by people with knee OA and to analyse its determinants, according to Andersen’s behavioural model. Secondarily, we aim to describe the overall healthcare services used by people with knee OA.

## Methods

### Data source

This study analyses the *EpireumaPt* project database, a national cross-sectional population-based study with a representative sample of the Portuguese population. *EpiReumaPt* aimed to develop a comprehensive understanding of the burden of Rheumatic and Musculoskeletal Diseases (RMD’s) in Portugal. As described in detail elsewhere [20], the *EpiReumaPt* study recruitment used a three-phase approach, over the period September 2011 to December 2013. The sample of *EpiReumaPt* study was recruited from a random selection of private households in Portuguese Mainland and Islands (Madeira and Azores), and was stratified according to the administrative territorial units [(NUTS II) (Norte, Centro, Lisboa and Vale do Tejo, Alentejo, Algarve, Açores Islands (Azores) and Madeira Islands (Madeira)], and the size of the population within each locality (< 2000; 2000–9999; 10,000–19,999; 20,000–99,999; and ≥ 100,000 inhabitants, respectively). In each household, an individual ≥18 years old with permanent residence and the most recently celebrated birthday was selected to be a participant in the study. Each selected household was visited, with no previous contact, up to three times, if no candidate participant was present during the first visit. In the long run, 28,502 households were contacted, 8041 individuals refused to participate in the study, and 10,661 were included. The *EpiReumaPt* population was similar to the Portuguese population (CENSUS 2011) in age strata, sex, and NUTII distribution [20].

In the first phase of the study, the participants completed a face-to-face interview to collect health-related information, which also screened for RMD’s, by a team of non-medical healthcare professionals trained for this purpose. The interviews were conducted using a Computer Assisted Personal Interview (CAPI) system. An individual was considered to have a positive screening if the subject mentioned a previously known RMD, if any of the algorithms in the screening questionnaires was positive, or if the subject reported muscle, vertebral or peripheral joint pain in the previous 4 weeks.

The overall performance of the screening algorithm was evaluated (the gold standard was considered the final diagnosis after revision - phase 3) and the overall sensitivity of the screening questionnaire for RMD’s was 98%, with a specificity of 22%. The positive predictive value was 85% and the negative predictive value was 71% [20].

**Fig. 1:**
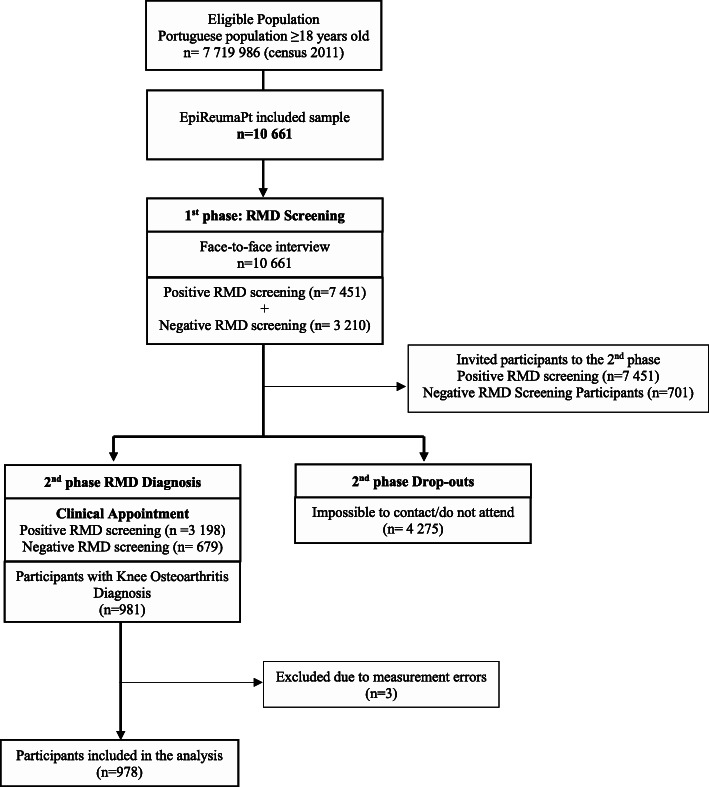
Flowchart of EpiReumaPt study design. *RMD, Rheumatic and Musculoskeletal Diseases*

The participants who screened positive for at least one RMD (*n* = 7451), as well as approximately 20% (*n* = 701) of participants with negative screening for RMD’s, were invited for a second phase, that consisted of a clinical appointment with a Rheumatologist. Of these, 4275 did not attend the clinical appointment. Therefore, at the end of phase 2 there were 3877 clinical observations: 3198 received the validation of RMD’S and 679 did not have an RMD diagnosis. The clinical assessments were performed at the Primary Care Centre of the participants neighbourhood, with a mobile van, fully equipped, to perform imaging and laboratory tests, supported by a multidisciplinary team with a rheumatologist, and X-Ray technician, a nurse, a staff coordinator and a driver. The clinical appointments consisted of a structured evaluation, laboratory and imaging exams, if needed, to establish the diagnosis and evaluate disease-related information. The rheumatologists involved were blind to the prior health-related data. In the third-phase, experienced rheumatologists reviewed all the data and confirmed the diagnosis – Fig. 1 [20]. When data was insufficient to fulfil the international classification criteria for each RMD, an additional meeting of the experts took place in order to discuss and reach an agreement on the final diagnosis. If at this stage no agreement had been reached, the opinion of the rheumatologist that performed the clinical assessment (second phase) prevailed. Diagnostic agreement between the 3 reviewers was 98.3% with a Cohen’s K coefficient of 0.87 (95%CI 0.83, 0.91. A total of 981 participants had a validated diagnosis of knee OA [20].

### Study population

This study includes the participants of *EpiReumaPt* with knee OA diagnosis, validated in the second phase of *EpireumaPt*, according to the American College of Rheumatology criteria: knee pain with at least three of the following clinical findings: age > 50 years, morning stiffness < 30 min duration, *crepitus* in active motion, tenderness of the bone margins of the joint, bony enlargement noted on examination, and lack of palpable warmth of the synovium [21].

### Outcomes

The healthcare services utilisation data is the outcome of interest, collected in the first phase of the study. Participants were asked if they had attended any medical appointments, undergone hospitalisations, surgery, psychology and physiotherapy consultations and, to indicate the number of appointments in the previous 12 months. The number of General Practitioner (GP) appointments was categorised in “no appointments”, “1–2 appointments” and “3 or more appointments”. The reason for hospitalisation was asked. Joint surgery was considered, although the presented variable does not distinguish between joint replacement surgery or other joint surgery. Based on the possible number of medical appointments and physiotherapy sessions within a year, and according to the distribution of number of medical appointments and physiotherapy sessions, the participants with > 60 medical appointments or > 180 physiotherapy sessions were considered to be an error of data insertion and were excluded (*n* = 3).

### Determinants

The determinant variables were collected during the first phase of *EpiReumaPt*, and are presented according to Andersen’s model, as previously described. Due to the low frequency of response in some of the original categorical variables, and to ensure a better interpretation of the results, some categories of categorical variables were amalgamated and some continues variables were transformed in categorical variables, as described in detail below.

#### Predisposing characteristics

Predisposing characteristics included: age, sex, geographic location, according to NUTS II territorial units and marital status, previously described [20]. Madeira and Azores were merged in the analysis as Island’s region The variable marital status was dichotomized into: “with partner”, which includes participants who are married or who live in a consensual union, and “without partner”, which includes participants who are single, widowed or divorced.

#### Enabling factors

These factors included: work status, firstly presented as employed (full or part time), retired, unemployed, incapable of working due to rheumatic disease and others (domestic worker, students, live with revenues) and then categorised as employed and non-employed (unemployed, retired, incapable of working due to rheumatic diseases and others); have or do not have healthcare insurance, additional to NHS coverage; number of years of schooling, that was categorised as having < 4 and ≥ 4 years of schooling, representing the attendance (or not) of at least the first stage of primary education.

#### Need variables

Need variables included the number of self-reported chronic comorbidities: high blood pressure, high cholesterol, cardiac disease, diabetes mellitus, chronic lung disease, problems in the digestive tract, renal colic, neurological disease, allergies, mental or psychiatric illness, cancer, thyroid and parathyroid problems, hypogonadism, hyperuricemia. The presence of other rheumatic diseases (excluding knee OA), diagnosed by the rheumatologists’ team, was added. Body mass index (kg/m^2^) was calculated with self-reported height and weight, and categorised as underweight (≤18.49 kg/m^2^), healthy weight (≥18.5 and ≤ 24.99 kg/m^2^), overweight (≥25 and ≤ 29.99 kg/m^2^) and obese (≥30 kg/m^2^). Lifestyle variables such as alcohol intake and smoking habits (both categorised as never, occasionally and daily) were noted, as well as past habits of smoking. Regular physical exercise/sports habits were also questioned (yes/no). Health-related quality of life (HRQoL) was assessed using EuroQol, with 5 dimensions and 3 levels (EQ-5D-3L) [22]. The index score ranges from 1, which represents full health, and zero or below that corresponds to death or states worse than death. Anxiety and depression symptoms were evaluated using the Hospital Anxiety and Depression Scale (HADS) for subscales of depression (HADS-D) and anxiety (HADS-A). Both fall into a range from 0 to 21, where higher values represent greater symptoms of anxiety or depression [23]. Physical function was measured based on the Health Assessment Questionnaire (HAQ). Where total scores lying between zero, indicating no functional impairment, and 3 indicating complete impairment [24]. The use of regular medication and number of medicines was also collected.

### Statistical analysis

All statistical analyses were performed using SPSS 24 for MacOS (IBM Corp., Armonk, NY, USA).

In the first stage, using descriptive statistic methods, we explored the health services most used by participants with knee OA. With the results of this analysis and the knowledge of the literature previously published in this field We included in the Two Step Cluster procedure (TSC) four variables: 1) number of GP appointments (no appointments, 1–2 appointments and ≥ 3 appointments); 2) orthopaedic specialist appointments (yes or no); 3) physiotherapy sessions (yes or no); 4) hospitalization (yes or no). The categorisation of the variable “GP appointments” was made according to the median value of the distribution of this variable in the sample (x = 3.00).

The TSC procedure is a hybrid approach that uses a distance measure to separate groups, and an agglomerative hierarchical clustering based on best fit to choose the optimal subgroup model. In this procedure, we used the Schwarz’s Bayesian information criterion (BIC) as a statistical measure of best fit to determine the number of clusters, the log-likelihood as distance measure and the average silhouette coefficient (ASC) as the silhouette measure of cluster cohesion and separation. We accepted the cluster solution considering the highest ratio of distance measure. Evidence shows that TSC is one of the most reliable procedures in terms of the number of subgroups detected, classification probability of individuals to subgroups and reproducibility of findings on clinical data [25].

We used descriptive statistics and non-parametric tests for independent samples (Kruskal-Wallis for continuous variables and chi-squared test for categorical variables, *p* < 0.05) to describe and compare the determinants and health utilisation in the entire sample and between clusters.

In addition, through a sensitivity analysis to ensure the robustness of our results, we also explored the association between the determinant variables and the cluster membership. First, we conducted a univariate analysis to select the variables for inclusion in the multinomial logistic regression model, with a significance level of 0.2, to avoid early exclusion of potential important variables [26]. Then, in the multinomial regression procedure, we performed a stepwise hierarchical analysis according to the domains of Andersen’s model in three steps: 1) inclusion of predisposing characteristics in the model and removal of non-significant variables; 2) inclusion of enabling factors in the previous model and removal of non-significant enabling factors; 3) inclusion of need variables in the previous model and removal of non-significant need variables, resulting in the final model. Odds-Ratio (OR) was estimated for each variable with 95% confidence interval (CI). Participants with missing data were automatically excluded from this analysis. This model was adjusted for sex and age, as important confounder variables for healthcare utilisation.

We evaluated the discriminative capacity of each model in each of the three steps calculating a binomial area under the receiver operating curve (AUC), to analyse the proportion of increment in the discriminative capacity in each step. The binomial AUC was calculated using the estimated classification probability for a given cluster, regarding the reference cluster. The discriminative capacity was considered weak if AUC was between 0.5–0.69; acceptable if between 0.7–0.79 and good if above 0.80. We also analysed the variance of the multinomial model using the McFadden Pseudo-R^2^ in each step [27].

### EpireumaPt ethical issues

*EpireumaPt* study was approved by the Ethics Committee of NOVA Medical School and by the Portuguese Data Protection Authority (Comissão Nacional de Proteção de Dados). Written informed consent was obtained from all participants, in accordance with the Declaration of Helsinki, as described elsewhere [20].

## Results

### Profiles of healthcare utilisation

Among the 978 participants diagnosed with knee OA included in the analysis, we found three different profiles of healthcare services utilisation with the TSC procedure, based on the healthcare services most used – Table 1. We named the clusters according to the attendance to orthopaedic surgeon appointments, physiotherapy sessions, number of GP appointments and hospitalisation.

1. “High Healthcare Users” (*HighUsers*): included all participants from the sample who had at least one appointment in the previous 12 months with the orthopaedic surgeon, who had physiotherapy and who had hospitalisation. In this cluster, the distribution of participants among the three categories of GP appointments was heterogeneous. The participants included in the *HighUsers* cluster represent 35.07% of the sample.
2. “GP users” (*GPUsers*): included only participants who had 3 or more GP appointments in the last 12 months and no use of the other services. Participants included in the *GPUsers* cluster represent 27.8% of the sample.
3. “Low healthcare users*”* (*LowUsers*): included participants who had less than 2 appointments with the GP in the previous 12 months, and no use of the other healthcare services. Participants included in *LowUsers* cluster represent 37.11% of the sample

**Table 1:** Healthcare utilisation of total sample and Clusters

	Total^**f**^ n (%) 978 (100)	***HighUsers*** n (%) 343 (35.07)	***GPUsers*** n (%) 272 (27.80)	***LowUsers*** n (%) 363 (37.11)	***p***-value
**Cluster Variables**^**a**^					
GP Appointments					
0	126 (12.9)	30 (8.7)	0 (0)	96 (26.4)	< 0.001^d^
1–2	379 (38.8)	112 (32.7)	0 (0)	267 (73.6)	
≥ 3	473 (48.4)	201 (58.6)	272 (100)	0 (0)	
Physiotherapy^b^	141 (14.4)	141 (41.1)	0 (0)	0 (0)	< 0.001^d^
Orthopaedic Surgeon^b^	192 (19.6)	192 (56.0)	0 (0)	0 (0)	< 0.001^d^
Hospitalisation^b^	112 (11.5)	112 (32.7)	0 (0)	0 (0)	< 0.001^d^
**Healthcare Use**					
GP appointments, mean ± SD	3.14 ± 3.32	3.38 ± 3.38	4.00 ± 1.17	1.27 ± 0.83	< 0.001^e^
Other medical appointments^b^					
Rheumatology	56 (5.7)	29 (8.5)	12 (4.4)	15 (4.1)	0.026^d^
Physiatry	43 (4.4)	39 (11.4)	4 (1.5)	0 (0)	< 0.001^d^
Cardiology	136 (13.9)	72 (21.0)	25 (9.2)	39 (10.7)	< 0.001^d^
Neurology	50 (5.1)	24 (7.0)	17 (6.3)	9 (2.5)	0.015^d^
Internal medicine	77 (7.9)	10.8 (37)	24 (8.8)	16 (4.4)	0.006^d^
Surgery	71 (7.3)	55 (5.6)	8 (2.9)	8 (2.2)	< 0.001^d^
Psychiatry	45 (4.6)	19 (5.5)	15 (5.5)	11 (3.0)	0.197^d^
Other medical appointments^c^, mean ± SD	2.41 ± 4.35	4.43 ± 6.65	3.82 ± 4.75	1.82 ± 2.70	< 0.001^e^
Joint Surgery^b^	20 (2.3)	20 (8.0)	0 (0)	0 (0)	< 0.001^d^
Psychology^b^	13 (1.3)	5 (1.5)	1 (0.4)	7 (1.9)	0.228^d^
Technical aids^b^	18 (1.8)	17 (5.0)	1 (0.4)	0 (0)	< 0.001^d^

Categorical variables are presented as n(%); continuous variables are presented as mean ± standard deviation

*GP* General Practitioner

^a^Variables included in the Two Step Cluster procedure.^b^At least once in the previous 12 months.^c^Number of visits related with the previous 12 months.^d^Chi-square test for independency; ^e^Kruskal-Wallis test

^f^Sample Size is not constant due to participants with validated data in the following variables: Joint Surgery (*n* = 885) and Technical aids (*n* = 973)

This cluster solution presents an ASC of 0.6, which shows a good model fit, and the ratio of distance measures was 1.706.

Regarding the total sample, 87.2% of the participants reported at least one GP visit, 14.4% were enrolled in physiotherapy, 19.6% had visited the orthopaedic surgeon and 11.50% were hospitalised in the last 12 months. *HighUsers* represent the participants with higher number of medical appointments (4.43 ± 6.65, *p* < 0.001) among the majority of medical specialities. *GPUsers* includes participants with a higher utilisation of GP appointments (4.00 ± 1.17, *p* < 0.001), and who take more regular medication (1.12 ± 3.86, *p* < 0.001).

### Characteristics of the sample and clusters

Women represent 73% of the sample, the mean age of participants was 65.34 (±11.30) years old, 247 (25.3%) participants have less than 4 years of education, and only 15% of participants were employed. The majority of the participants are overweight (41.8%) or obese (40.8%) and only 20.6% report doing regular physical exercise. Distributions across clusters were statistically different (*p* < 0.05) for the majority of predisposing characteristics, enabling factors and need variables – Table 2.

**Table 2:** Predisposing characteristics, enabling factors and need variables distribution of total sample and clusters

	Determinants	Total^**a**^ n (%) 978 (100)	***HighUsers*** n (%) 343 (35.07)	***GPUsers*** n (%) 272 (27.80)	***LowUsers*** n (%) 363 (37.11)	*p-value*
Predisposing	Age	65.34 ± 11.30	65.18 ± 10.51	66.47 ± 10.35	64.74 ± 12.02	0.081^b^
	Female sex	714 (73)	259 (75.5)	209 (76.8)	246 (67.8)	0.017^c^
	Geographic Location					
	North	255 (29.3)	105 (34.0)	60 (28.6)	81 (25.2)	< 0.001 ^c^
	Centre	243 (27.9)	85 (27.2)	87 (36.1)	72 (22.4)	
	Islands	127 (14.6)	32 (10.4)	21 (8.7)	74 (23.1)	
	South	84 (9.6)	29 (9.4)	26 (10.8)	29 (9.0)	
	Lisbon	162 (18.6)	59 (19.1)	38 (15.8)	65 (20.2)	
	Married or consensual union	619 (63.3)	222 (64.7)	165 (60.7)	232 (63.9)	0.556 ^c^
Enabling	Years of Education	4.96 ± 3.32	5.05 ± 3.36	4.36 ± 3.18	5.32 ± 3.32	< 0.001^b^
	< 4 years of Education	247 (25.3)	79 (23.1)	98 (36.0)	70 (19.3)	< 0.001^c^
	Employment status					0.006 ^c^
	Employed (part-time OR full-time)	145 (16.6)	42 (13.5)	31 (12.9)	72 (22.9)	
	Retired	634 (72.4)	222 (71.4)	185 (76.7)	228 (70.2)	
	Unemployed	68 (7.8)	31 (10.0)	18 (7.5)	19 (5.8)	
	Temporarily disabled	17 (1.9)	11 (3.5)	4 (1.7)	2 (0.6)	
	Others^d^	12 (1.4)	5 (1.6)	3 (1.3)	4 (1.2)	
	NHS as only health system	763 (78.0)	236 (74.9)	230 (79.3)	297 (79.6)	0.003^c^
Need	Physical function (HAQ)	0.76 ± 0.69	0.88 ± 0.66	0.77 ± 0.67	0.47 ± 0.59	< 0.001^b^
	HRQoL (EQ-5D-3L)	0.62 ± 0.27	0.57 ± 0.27	0.61 ± 0.26	0.73 ± 0.25	< 0.001^b^
	BMI (kg/m^2^)	29.54 ± 5.06	29.71 ± 4.77	29.69 ± 5.14	29.27 ± 4.95	0.366^b^
	Underweight (< 18.5 kg/m^2^)	2 (0.2)	0 (0)	0 (0)	2 (0,6)	0.311^c^
	Normal weight (18.5–24.99 kg/m^2^)	156 (17.2)	59 (15.2)	40 (16.1)	67 (19.9)	
	Overweight (25–29.99 kg/m^2^)	379 (41.8)	135 (41.8)	110 (44.4)	134 (39.9)	
	Obese (≥30 kg/m^2^)	370 (40.8)	139 (43.0)	98 (39.5)	133 (39.6)	
	Anxiety (HADS-A)	6.86 ± 4.15	7.08 ± 4.20	7.54 ± 4.23	5.85 ± 3.94	< 0.001^b^
	Depression (HADS-D)	6.09 ± 4.30	5.74 ± 3.96	6.59 ± 4.21	4.94 ± 3.82	0.001^b^
	Number of Comorbidities	4.47 ± 2.44	4.48 ± 2.42	5.11 ± 2.54	3.65 ± 2.19	< 0.001^b^
	Daily alcohol intake	199 (20.4)	70 (20.5)	53 (19.5)	76 (20.9)	0.077^c^
	Active smoker	71 (7.3)	25 (7.3)	16 (5.9)	30 (8.3)	0.519^c^
	Ex-smoker	182 (17.9)	59 (18.6)	38 (14.8)	65 (19.5)	0.312^c^
	Regular physical activity	201 (20.6)	58 (17.0)	41 (15.1)	102 (28.1)	< 0.001^c^

Categorical variables are presented as n (%); continuous variables are presented as mean ± standard deviation

*NHS* National Health System, *GP* General Practitioner, *HRQoL* Health Related Quality of Life, *EQ-5D-3L* EuroQol with five dimensions and three levels, *HAQ* Health Assessment Questionnaire, *HADS-A* Hospital Anxiety and Depression Scale – Anxiety subscale, *HADS-D* Hospital Anxiety and Depression Scale – Depression subscale

^a^Sample size is not constant due to participants with validated data in the following variables the following: Geographic location (*n* = 871), Years of education (*n* = 977), Employment status (*n* = 876), EQ-5D-3L score (*n* = 965), Alcohol intake (*n* = 977), BMI (*n* = 971), Regular physical activity (*n* = 977)

^b^Kusskall-Wallis test

^c^ Chi-squared test

^d^This category includes participants who are students, domestic workers or that lives on revenues

### Determinants of cluster membership

After the univariate analysis (Supplementary file), variables at < 0.2 significance level were considered for the multinomial logistic regression model. The reference category was *LowUsers* cluster. Due to missing data, 146 (14,93%) participants were excluded from this analysis, but the proportion of excluded participants was similar between clusters – Table 3.

**Table 3:** Final Multinomial Regression Model

		HighUsers vs. LowUsers			GPUsers vs. LowUsers		
Variables added	Determinants	OR	95%CI	***p***-value	OR	95%CI	***p***-value
**Predisposing Characteristics**	Age	0.96	0.95–0.99	0.001	0.99	0.96–1.01	0.172
	Male sex^a^	1.05	0.69–1.58	0.826	1.12	0.72–1.76	0.613
	Geographic Location^b^						
	*North*	1.545	0.93–2.56	0.091	1.23	0.70–2.15	0.475
	*Centre*	1.50	0.89–2.51	0.130	2.11	1.21–3.68	0.008
	*Islands*	0.43	0.24–0.77	0.005	0.42	0.21–0.83	0.013
	*South*	1.49	0.76–2.94	0.250	1.82	0.89–3.75	0.102
*Step 1*	**AUC**^g^	0.58	0.54–0.61	0.001	0.61	0.57–0.65	0.001
	**McFadden Pseudo-R**^**2**^ **= 0.026**						
**Enabling Factors**	NHS only^c^	0.65	0.43–0.98	0.042	1.34	0.81–2.22	0.249
	< 4 Years of Education^d^	0.90	0.56–1.45	0.900	1.50	0.93–2,43	0.096
	Employed^e^	0.55	0.31–0.97	0.038	0.81	0.44–1.50	0.512
*Step 2*	**AUC**^g^	0.60 (+ 0.02)	0.56–0.64	0.001	0.63 (+ 0.02)	0.59–0.67	0.001
	**McFadden Pseudo-R**^**2**^ **= 0.040 (+ 0.014)**						
**Need Variables**	Number of Comorbidities	1.12	1.03–1.21	0.011	1.22	1.11–1.33	< 0.001
	HRQoL (EQ-5D-3L index score)	0.33	0.14–0.79	0.013	0.61	0.23–1.57	0.303
	Physical function (HAQ score)	1.59	1.10–2.23	0.013	1.03	0.69–1.53	0.902
	Anxiety (HADS-A)	1.02	0.97–1.07	0.432	1.09	1.03–1.14	0.002
	Regular Physical Exercise^f^	0.57	0.37–0.88	0.010	0.55	0.34–0.89	0.014
*Step 3: Final model*	**AUC**^g^	0.68 (+ 0.08)	0.64–0.71	0.001	0.69 (+ 0.07)	0.65–0.73	0.001
	**McFadden Pseudo-R**^**2**^ **= 0.098 (+ 0.058)**						

*NHS* National Health System, *GP* General Practitioner, *HRQoL* Health Related Quality of Life, *EQ-5D-3L* EuroQol with five dimensions and three levels, *HAQ* Health Assessment Questionnaire, *HADS-A* Hospital Anxiety and Depression Scale – Anxiety subscale. Reference Categories: ^a^Female; ^b^Lisbon and Tagus valley; ^c^ Healthcare insurance along with NHS; ^d^ ≥ 4 years of education; ^e^Non employed or retired; ^f^ Don’t perform regular physical exercise; ^g^Area Under the ROC Curve (95% CI) – reference cluster is LowUsers. Differences in discriminatory capacity (AUC) and in variance of the model regarding the previous step is shown in brackets. χ^2^(28) = 180.328, *p* < 0.001

This procedure excluded all the participants with missing data. Sample included in the analysis: Total: *n* = 838 (85,69% of the initial sample), HighUsers: *n* = 295 (86.0% of the initial cluster sample); GPUsers: *n* = 232 (85,29% of the initial cluster sample); LowUsers: *n* = 311 (85.67% of the initial cluster sample)

As seen in the Tables 3 and 4, in the multinomial logistic model, having *LowUsers* as the reference cluster, the following determinants were associated with *HighUsers* cluster membership: being younger (OR = 0.96, 95% CI 0.95, 0.99) and reside in Portugal mainland, when compared to reside on islands (OR = 0.43, 95% CI 0.24, 0.77) as predisposing characteristics; have additional health coverage (OR = 0.65, 95% CI 0.43, 0.98) and being employed (OR = 0.55, 95% CI 0.31–0.97) as enabling factors; and higher number of comorbidities (OR = 1.12, 95%CI 1.03, 1.21), worse HRQoL (OR = 0.33, 95% CI 0.14, 0.79), worse physical function (OR = 1.59, 95% CI 1.10–2.23) and no regular physical exercise (OR = 0.57, 95% CI 0.37, 0.88) as need variables. The only predisposing characteristic associated with *GPUsers* membership was geographic location. Residing in the centre when compared to reside in Lisbon region (OR = 2.11, 95% CI 1.21, 3.68), and in Portugal mainland when compared to reside in the Islands region (OR = 0.42, 95% CI 0.21, 0.83), increase the probability of being classified as *GPUser,* with *LowUsers* as the reference cluster. No enabling factors had statistical association within *GPUsers* cluster membership. Higher number of comorbidities (OR = 1.22. 95% CI 1.11, 1.33), the presence of anxiety symptoms (OR = 1.09, 95% CI 1.03, 1.14) and have no regular physical exercise (OR = 0.55 95% CI 0.34, 0.89) were the need variables associated with *GPU* cluster membership. A higher variation in the AUC and in the McFadden pseudo-R^2^ occurred when need variables were entered in the model.

**Table 4:** Summary of determinants that increase the probability of membership in each healthcare utilisation profile, according to Andersen’s Behaviour Model of Healthcare Utilisation

	Determinants	***HighUsers***^***a***^	***GPUsers***^***a***^
**Predisposing Characteristics**	Age	Being Younger	–
	Geographic Location	Live in Portugal Mainland	Live in Portugal Mainland Live in the centre region
**Enabling Factors**	Healthcare insurance	Additional healthcare coverage	–
	Employment status	Being employed	–
**Need Variables**	Comorbidities	Higher number of comorbidities	Higher number of comorbidities
	Quality of life	Worse HRQoL	–
	Physical function	Worse physical function	–
	Anxiety symptoms	–	More anxiety symptoms
	Physical Exercise	No regular physical exercise	No regular physical exercise

*NHS* National Health System, *HRQoL* Health Related Quality of Life

^**a**^Reference Cluster: LowUsers

## Discussion

### Healthcare services utilisation in Portugal

In this study, we identified three profiles of healthcare utilisation according to the services most used by the participants with knee OA. The profile with the highest healthcare utilisation – *HighUsers,* represents more than 35% of the sample and was characterised by participants with appointments with the GP, orthopaedic surgeon, physiotherapy sessions and/or with hospitalisation. Given the high number of other medical appointments, this profile is possibly responsible for a high proportion of the total costs spent with people with knee OA in Portugal. As Warwick et al. [28] concluded, analysing an insurance database with more than 40,000 of people with knee OA, the top 30% of high-payment patients with OA accounted for more than 70% of overall non-arthroplasty payments.

Primary care is considered the most relevant setting for prevention and management of knee OA, where the conservative non-pharmacological interventions should be considered early, and throughout the progression of the disease [8, 9]. However, in our sample, few participants were enrolled in physiotherapy or regular exercise programmes and a high proportion were overweight. The study of Østeras et al. [29] found similar data, when analysing a sample of Portuguese people with knee OA in primary healthcare: only 20% of participants were referred to weight management programmes, and only 43% were referred to physical exercise programmes, in a similar fashion to other European countries included. However, in our sample, the proportion of participants who had undergone physiotherapy treatments (14.4%) was much lower compared to the 39–52% observed, for example, in the UK [30]. Overall, this data may suggest a weak adoption of the core recommended interventions for the management of knee OA, and possibly, be responsible for suboptimal outcomes and higher health costs, in Portugal. Moreover, Bedard et al. [31] estimated that if health professionals followed current clinical practice guidelines, the non-inpatient costs with OA would decrease by 45%. This data should sufficiently alarm health politicians regarding the need for the implementation of effective and recommended modalities in the management of people with knee OA at a national level.

### Determinants for healthcare services utilisation

Overall, the characteristics of our sample are similar to other data related to people with multimorbidity and the older adult population in Portugal, namely given the high proportion of people with lower education, high proportion being overweight or obese, and physically inactive [5, 32].

Our findings show that, regardless of clinical need, predisposing characteristics and enabling factors such as age, geographic location, health insurance and employment status, play an important role in healthcare utilisation. This data may disclose that, possibly, the current management of knee OA is heterogenous, not consistent with the needs of the patients, and also, highlights possible inequities in the access of health care [18].

In our analysis, younger and employed participants were positively associated with *HighUsers* profile. Unlike the data related to general older adults population in Portugal [33], evidence suggests that older adults with knee OA are less likely to be referred to specialised services, like an orthopaedic surgeon, rheumatologist [34] or to physiotherapy [30]. Qualitative data suggests that GP’s often consider OA as a normal consequence of ageing, attributing low importance to this condition in older adults [35]. In contrast, knee OA is associated with work-related disability, absenteeism, early retirement, psychological distress and low HRQoL in younger patients [4, 36]. Thus, employed or younger adults with knee OA seem to behave more proactively in seeking help and their physical limitations are generally taken more seriously by GP’s, with higher referral rates and consequently, a higher utilisation of healthcare services [35].

Our findings also suggest that geographic location is a determinant to healthcare services utilisation, namely the Islands and Centre region. Both of these regions are far from city centres, with higher proportion of older, less educated and poorer people. These regions experience a shortage of medical specialists such as orthopaedic surgeons. Moreover, Madeira and Azores are underserved by primary care units [37]. International data suggests that the distance from healthcare units, lack of transport and consequent isolation, and the perception of OA as being a self-limited condition, may prevent people from rural areas of seeking healthcare services timely, with lower healthcare resources utilisation as consequence [38, 39].

Participants with additional healthcare coverage were more likely to be *HighUsers*, suggesting that the NHS may not provide optimal access to the appropriate interventions according to the patients’ needs, or that the facilitation of access to private sector may enhance the utilisation of healthcare services, regardless of the severity of the disease [40]. In accordance with our study, private health insurance was the most frequently cited enabler in Australia for surgical and conservative OA treatments, such as physiotherapy [41].

Overall, our findings suggest that the delivery of healthcare for Portuguese people with knee OA may be inefficient and unfair, where people with better predisposing and enabling features consume a higher amount of healthcare services, than people without those features. Our findings, with the support of the presented literature, should raise concerns regarding the need to tackle health access inequities in Portugal. In this way, the organisation of the health system should guarantee that people with OA receive effective interventions according to clinical severity, and not according to sociodemographic factors.

For predisposing variables, our findings showed that the number of comorbidities is associated with higher healthcare utilisation profiles, mainly with *GPUsers* profile, as well as anxiety symptoms. People with OA visit primary care mostly in case of multimorbidity [42]. However, evidence shows that, in people with OA and multimorbidity, joint pain is often seen as a low priority problem, brought up late in the consultation, with low referral rates to physiotherapy or specialised care targeted to OA [42, 43]. This information may explain the stronger association of number of comorbidities with *GPUsers* profile, than with *HighUsers*. Regarding anxiety symptoms, contradictory data was found in literature. Anxiety is associated both negatively and positively with the utilisation of healthcare services [44, 45]. However, it is well known that mental health comorbidities, like anxiety and depression, as well as cardiovascular and metabolic comorbidities are associated with higher severity symptoms and poor outcomes in people with OA [45, 46]. Thus, the management of people with OA, especially with anxiety and/or multimorbidity, should be multidisciplinary personalised and targeted [8, 9], which would justify a higher utilisation of healthcare, mostly specialised services, partly in contrast to our data. Thus, we may argue that this subpopulation of patients with knee OA is undertreated in Portugal, recognising the urge to organise services across healthcare sectors to pursue the delivery of recommended and more effective interventions, mainly to people with poor prognosis.

In our study, physical inactivity was associated with both profiles of higher healthcare utilisation. Sedentary behaviour and being overweight in people with knee OA is associated with poor physical function, higher risk of cardiovascular comorbidities [7], higher healthcare consumption and higher health-related costs [47]. Barriers to physical exercise have been identified in literature that justify the low adherence of patients, namely the misbeliefs of health professionals regarding exercise and physiotherapy [48].

As expected, low levels of physical function and HRQoL are associated with *HighUsers*. A 10-year UK survey reported that disability was the strongest predictor for referral to specialised care and for TKR in people with knee pain [34]. Similarly to our data, poor physical function, associated comorbidities, and also radiologic severity were also associated with higher direct and indirect costs as reported in a Spanish survey [49]. Considering physical function and quality of life, the results of this study suggest that a higher healthcare utilisation does not reflect better outcomes.

### Strengths and limitations

This is the first study in Portugal analysing the health services utilisation by people with knee OA at a national level. The large sample, the multi-domains of the dataset and its framing on An dersen’s model, provides a comprehensive view of the current healthcare utilisation profiles and its determinants.

Nevertheless, it has some limitations. The cross-sectional design does not does not allow the establishment of a temporal relationship between determinants and healthcare utilisation; thus, cause and effect can be overestimated mainly in modifiable variables like physical function or HRQoL. Other potential important psychosocial variables, that may influence healthcare utilisation behaviours were not controlled in this study (e.g., coping behaviour, self-efficacy) [50]. The physical activity variable did not take into account the amount of time spent per week, nor its intensity, thus our results may be, even so, overestimated when comparing to the recommendations for physical activity. Public or private appointments, were not distinguished, which could increase the importance of predisposing characteristics and enabling factors in the variance of healthcare utilisation. As self-reported healthcare utilisation is related to the previous 12 months, we acknowledge that the possibility of memory bias may compromise the accuracy of the outcome of this study (utilisation of healthcare services). In this study, we did not account for the reason for medical appointments or physiotherapy attendance, which could increase the accuracy of the results. The data used was collected in 2011–2013 but, due to the few specific strategies directed to musculoskeletal diseases in the last decade in Portugal, we cautiously believe that the actual management of OA does not differ from this study.

### Implications of the findings

The results of this study highlight the importance of addressing the inequalities of access and heterogeneity in care, as well as the need to tackle adherence to exercise and enhancement of self-management strategies, e.g., with physiotherapy in primary care, to a much larger proportion of the population with knee OA. A whole system approach needs to consider primary prevention, early detection, cost-effective interventions and appropriate referral, as well as personalised interventions taking into account other comorbidities that are often present in these patients [51].

## Conclusion

We identified three different healthcare services utilisation profiles. The *HighUsers* profile accounted for more than one third of people with knee OA and it includes GP utilisation, orthopaedic surgeon appointments, physiotherapy and/or hospitalisation. Need variables explained a considerable proportion of the variance in healthcare utilisation, although determinants like younger age and geographic location, having additional healthcare coverage and being employed were associated with higher utilisation of healthcare services. These facts suggest the need for improvement in the access of healthcare services, in the quality of care and, implementation of international recommendations according to the clinical severity in all people with knee OA.

## Supplementary Information

**Additional file 1 **: **Table S1.** Univariate association analysis between the determinant variables and cluster membership.

## Data Availability

The data underlying this article were provided by the EpiDoc Unit - CEDOC by permission. Data will be shared on request to the corresponding author with permission of the EpiDoc Unit group leaders.

## Abbreviations

BMI: Body Mass Index
CI: Confidence Interval
EQ-5D-3L: EuroQol Questionnaire with 5 dimensions and 3 levels
GBP: Gross Domestic Product
GP: General Practitioner
GPUsers: General Practitioner Users profile
HADS-A: Hospital Anxiety and Depression Scale – Anxiety subscale
HADS-D: Hospital Anxiety and Depression Scale – Depression subscale
HighUsers: High Healthcare Users profile
HRQoL: Health Related Quality of Life
LowUsers: Low Healthcare Users profile
NHS: National Health Service
OA: Osteoarthritis
OR: Odds Ratio
TKR: Total Knee Replacement
