# Supplementary material for: Driving factors for the utilisation of healthcare services by people with osteoarthritis in Portugal: results from a nationwide population-based study
Source: BMC Health Serv Res. 2021 Sep 28;21:1022. doi: 10.1186/s12913-021-07045-4 (PMC8479902; doi:10.1186/s12913-021-07045-4)
Supplement: Supplementary file 1 — Additional file 1 : Table S1. Univariate association analysis between the determinant variables and cluster membership. [file 12913_2021_7045_MOESM1_ESM.docx]

| **Table S1 – Univariate association analysis between the determinant variables and cluster membership** | | | | |
| --- | --- | --- | --- | --- |
|  | **Determinants (eligible participants)** | **Chi-square** | **DF** | **p-value** |
| **Predisposing**  **Characteristics** | Age (n=978) | 3.99 | 2 | 0.136 |
|  | Sex (n=978) | 8.08 | 2 | 0.018 |
|  | *Geographic Location* (n=871) | 39.53 | 8 | <0.001 |
|  | Marital Status (n=978) | 1.16 | 2 | 0.558 |
| **Enabling Factors** | Healthcare Insurance (n=978) | 12.63 | 2 | 0.002 |
|  | Years of Education (n=977) | 23.60 | 2 | <0.001 |
|  | Employed (n=964) | 11.05 | 2 | 0.004 |
| **Need Variables** | Number of Comorbidities (n=978) | 55.20 | 2 | <0.001 |
|  | Body Mass Index (n=907) | 7.64 | 6 | 0.266 |
|  | HRQoL (EQ-5D-3L index score) (n=965) | 56.28 | 2 | <0.001 |
|  | Physical function (HAQ score) (n=978) | 63.67 | 2 | <0.001 |
|  | Depression (HADS-D) (n=978) | 22.09 | 2 | <0.001 |
|  | Anxiety (HADS-A) (n=978) | 31.84 | 2 | <0.001 |
|  | Physical Exercise (n=977) | 19.92 | 2 | <0.001 |
|  | Alcohol Intake (n=977) | 8.41 | 4 | 0.078 |
|  | Smoking Habits (n=978) | 1.34 | 2 | 0.512 |
| DF, degrees of freedom; HRQoL, Health Related Quality of Life; EQ-5D-3L, EuroQol with five dimensions and three levels; HAQ, Health Assessment Questionnaire; HADS-D, Hospital Anxiety and Depression Scale – Depression subscale; HADS-A, Hospital Anxiety and Depression Scale – Anxiety subscale  Only variables at <0.2 significant level were considered for inclusion in the multinomial logistic regression analysis. | | | | |
